# The Influence of Family Supportive Supervisor Behavior on Employee Creativity: The Mediating Roles of Psychological Capital and Positive Emotion

**DOI:** 10.3389/fpsyg.2022.824840

**Published:** 2022-05-12

**Authors:** Xiaogang Zhou, Liujun Jin, Yimeng Wang, Wenqin Liao, Honglei Yang, Liqing Li

**Affiliations:** ^1^School of Economics and Management, East China Jiaotong University, Nanchang, China; ^2^School of Economics and Management, Jiangxi Science and Technology Normal University, Nanchang, China

**Keywords:** family supportive supervisory behavior, creativity, psychological capital, positive emotions, structural equation model

## Abstract

In an increasingly complex external environment, innovation is an important way for companies to build sustainable competitiveness. This research discusses employee creativity from the perspective of Family Supportive Supervisor Behavior (FSSB) based on conservation of resource theory, social exchange theory, psychological capital theory and emotional spillover theory. Through a series of surveys of employees in different companies and jobs, we can understand the impact of family-supporting supervisors’ behavior on their creativity. Combined with the survey data, a structural equation model (SEM) is constructed to analyze the mediating effects of psychological capital and positive emotions based on the causal mediation model. The research found that the positive influence of family-supporting supervisors’ behavior on employees’ creativity has three forms. First, supervisors improve employees’ motivation and sense of efficacy by providing various support resources. Second, supervisors can generate positive spillover effects among employees by influencing employees’ psychological state. Third, supervisors stimulate the creativity of subordinates by promoting work participation and mobility. According to the research conclusions, in order to improve the employee creativity, we should provide incentives to encourage supervisors to carry out family support behaviors, identify employee characteristics to help supervisors provide personalized support, cultivate family supportive leaders, and attach importance to emotional support and play the role of psychological capital and positive emotions.

## Introduction

Nowadays, artificial intelligence, cloud computing and other technologies have been widely infiltrated into people’s daily work and life. As a result, the way of work styles is changing rapidly. The environment faced by organizations is becoming increasingly complex. The conflicts between work and family of employees continue to intensify. In this regard, managers generally begin to pay attention to the benefits of Family Supportive Supervisor Behavior (FSSB) in organizational governance (Hammer et al., 2013). Scholars such as Rofcanin et al. (2017) point out that FSSB was originally intended to solve work-family conflict and help employees balance the relationship between work and family affairs. FSSB not only has significant positive correlation with marital satisfaction, work-family conflict bidirectional and satisfaction, employee assistance and innovation behavior (Zhu Y. et al., 2015; Xie et al., 2017; Nie and Xie, 2018; Wang et al., 2018). At the same time, FSSB adopted by supervisors can relieve anxiety, help employees resolve conflicts in the work and family field, and enhance the sense of belonging of employees (Wang et al., 2020; Wang and Zhao, 2020). Niswaty et al. (2021) also proved the connection between work-family needs, supervisor support and role satisfaction through a longitudinal study of the Chinese context.

Innovation plays an important role in the technological and industrial revolution. Nowadays, creativity occupies a certain position in all fields of human social development, scientific and technological progress, and was even once known as the source of sustainable development in all fields (Sacramento et al., 2013; Qu and Liao, 2014; Leroy et al., 2015; Wang and Ye, 2015). Meanwhile, innovation behavior has gradually become an important part of employees’ work process (Thomas and Ganster, 1995). Many scholars have explored the stimulating mechanism of creativity from different perspectives. For example, positive emotions can effectively stimulate individual creativity (Tang et al., 2011). The input of psychological capital has positive effect on individual creativity (Chen et al., 2013). Leadership motivation is a powerful factor to stimulate employee creativity (Jiang and Yang, 2014; Ma and Wang, 2015; Wang and Chen, 2018). Unlike previous studies on the relationship between FSSB and employee creativity, mostly of which used behavioral theory of leaders, this study is based on conservation of resources theory, social exchange theory psychological capital theory and emotional spillover theory, assuming that psychological capital and positive emotions mediating variables in the relationship between FSSB and employee creativity.

Based on conservation of resource theory and social exchange theory, this study deeply explores the internal mechanism of the impact of FSSB on employees’ creativity with psychological capital and positive emotions as intermediary variables. In addition, this research investigates and analyses the supervisor support behavior and creativity received by the individual. Then it proposes countermeasures and suggestions with strong operability in order to help the company to reasonably and efficiently display the creativity of employees. The advantages of this study may be as follows: First, it helps enrich the understanding and provides new ideas of the relationship between FSSB and employee creativity from the perspective of resource conservation theory, social exchange theory, psychological capital theory, and emotional spillover theory. Second, it helps to verify the mediating role of positive emotion and psychological capital, makes up for the lack of simultaneous verification of the mediating role of the two variables between FSSB and employee creativity in the existing literature, lays a theoretical and empirical foundation for future exploration of work-family gain.

## Hypothesis Development and Research Model

### Effect of Family Support Supervisor Behavior on Employee Creativity

According to the Conservation of Resources Theory (COR; Hobfoll, 1989), employee creativity behavior is an activity with high resource consumption and strong intrinsic motivation. It will consume resources to a certain extent and require employees to continuously invest time and cognitive resources (Zhang and Bartol, 2018). In order to make employees actively engage in creative behaviors, it is necessary to enrich their own resources. First of all, FSSB can reduce the loss of resources caused by negative events such as family pressure and work-family conflict that employees face. It also helps employees perform family responsibilities more easily, reduce stress and thus be more creative (James, 2011; Van dyne et al., 2012). Secondly, FSSB enriches employees’ resources, which is manifested in psychological capital and positive emotions of employees and allows employees to have more resources for creativity (Dreisbach and Goschke, 2014; Aleksic et al., 2017). Finally, FSSB is more conducive to the formation of a safe work environment, which reduces the threat of resource loss in the Working Atmosphere and enables employees to actively exert their creativity (Carmeli and Russo, 2018).

The influence of different leadership behaviors on employee creativity is not always direct. Therefore, scholars often study from different perspectives when studying the relationship between them. Research by Liu et al. (2016) shows that leaders are the most direct factors in the workplace and have an important guiding influence on the development of employees’ core creativity. The empirical research results by Wang et al. (2016) show that individuals often need to invest enough resources when generating new ideas and expect external support and encouragement for their creative activities. FSSB provides the right context for employees to think that innovation makes sense and come up with new ideas. Sanjeet et al. (2021) points out that in order to maintain a stable and long-term relationship, individuals are more willing to do stylized work than to propose new ways, because employees are more sensitive to the risks inherent in creativity. However, individuals exhibit more creativity to meet organizational expectations when they perceive the encouragement and support of the organization accompanied by respect and reward. Luqman et al. (2021) points out that employees’ creativity plays an important role in the work field. Especially in the current business environment, the continuous cultivation of this thinking ability plays a significant role in the long-term development of enterprises. Wang et al. (2021) believes that the endogenous of individual creativity is inseparable from active and effective supervisor behavior. As a people-oriented and family-friendly cultural policy, FSSB is a powerful embodiment of positive leadership behavior, which is bound to have a positive impact on promoting employee creativity. Based on the above research, this article proposes the following hypothesis:

**Hypothesis 1:** Family supportive supervisor behavior positively affects employee creativity.

### Effect of Family Supportive Supervisor Behavior on Psychological Capital

Psychological capital, as a positive psychological state, is also an individual’s positive internal resource (Zhao and Gao, 2014). Psychological capital includes self-efficacy, hope, optimism, and resilience. In essence, psychological capital is the psychological resource that promotes personal growth and performance improvement. According to the resource preservation theory, when individuals have sufficient resources, they are less threatened by resource loss and more able to obtain resources. Supervisor’s support is an important resource for employees to carry out their work. FSSB can help to create a positive, healthy and sunny psychological state, provide a good working environment for employees and play an important role in increasing employees’ psychological capital. When employees feel the supervisor’s recognition and active concern for employees’ needs, their positive emotions such as self-efficacy and optimism are significantly improved. Kumari and Renu (2019) points out that people tend to pay more attention to inner satisfaction, so people hope to keep in touch with those who meet their psychological needs and exchange their resources, respectively.

In recent years, the development of positive psychology has been changing with each passing day. The shift from negative conflict to positive gain is a major change in the field of research on outcome variables of work-family relationships. For example, Luthans and Broad (2020) takes psychological resources and visual resources as Mediating variables and conducts an in-depth empirical study on the direct influence and effect of family-supportive supervisor behavior on employees’ work-family gain. Hao et al. (2020) points out that effectively enhancing employees’ psychological capital is the key to achieving results of various organizational support behaviors. Based on the social exchange theory, Huang and Wang (2021) points out that in the process of interpersonal communication, both parties of the group exchange activities with each other in order to obtain some of the other’s resources. The essence of social exchange is the result of mutual benefit and reciprocity. Based on the above research, this article proposes the following research hypothesis:

**Hypothesis 2:** Family supportive supervisor behavior positively affects employees’ psychological capital.

### Effect of Family Supportive Supervisor Behavior on Positive Emotions

Human mood or emotion are usually described by dichotomy (Diener et al., 1985). In subsequent studies, scholars usually use positive emotions and negative emotions to describe the different emotions of individuals. Waston and Clark (1984) believe that positive emotion is mainly reflected in the degree to which individuals can feel positive emotions. Lazarus (1991) believes that positive emotion is a pleasant feeling generated by individuals when they complete the target task and are recognized by the outside world. According to the emotion spillover theory, emotions generated in one role may cause spillover effect on another role, which can be divided into positive spillover and negative spillover (Edwards and Rothbard, 2000). FSSB can improve employees’ positive emotions through affective support, instrumental support and role model behavior.

Zhen et al. (2015) believe that it is necessary to bring positive emotions into the study of employees’ work-family relationship. They also point out there are two feedback paths in the model, one of which is called emotion path, and the important variable is positive emotions. Cai et al. (2018) find that “understanding,” “support” and other behaviors have an impact on employees’ emotions through positive emotions. Levasseur et al. (2020) believes that individuals who perceive support from supervisors are prone to endogenous positive emotions, assess risks optimally and take high-uncertainty work as an opportunity. Most theoretical research perspectives include the premise that “emotion plays an important role at the work-family level,” which also indicates that such emotion exists in the positive relationship between work and family. Based on the above research, this article proposes the following research hypothesis:

**Hypothesis 3:** Family supportive supervisor behavior employees’ positive emotions.

### The Mediating Role of Psychological Capital

With the emergence of positive organizational behavior, the research on its influencing factors has become “the theoretical growth point of constant appeal.” According to the resource conservation theory, people always try their best to maintain, protect and expand the valuable resources that they consider to be core values of themselves. Moreover, according to the social exchange theory, the supervisor is the embodiment of the organization and the family-supportive supervisor behavior is an important manifestation of the organization’s commitment to employees (Kottke and Sharafinski, 1988).

According to the principle of reciprocity norm, employees will have a stronger willingness to repay the organization after they perceive the support of supervisors, thus generating a closer exchange relationship between the organization and employees, so that employees can devote more energy to innovation (Zhu et al., 2015). In order to reduce the loss of resources due to innovative behaviors, these employees with lower psychological capital often choose a more conservative approach, while other employees with higher psychological capital believe that their creativity will bring lower losses to themselves and have a positive attitude toward the acquisition of new resources.

Ye et al. (2014) emphasize that employees can adjust their cognition of innovative work through supervisor supportive behavior, acquire knowledge, skills, experience and positive emotions required by their responsibilities, continuously self-reinforce, thus improving their psychological capital and seeking new ways to complete tasks. Wang et al. (2017) verify the positive impact of psychological capital on employee creativity and find the mediating effect of psychological security from the perspective of psychological capital and planned behavior. Han and Yang (2017) conduct a paired survey on leaders and employees in electric power enterprises, they conclude that family-supported leadership can effectively enhance employees’ psychological capital, thus stimulate employees’ creativity through the intermediary role of psychological capital. Miao et al. (2021) considers that supportive leadership behavior has a significant impact on positive emotions, which can improve individuals’ sense of control and sense of competence at work, make employees feel the significance of work and stimulate work motivation. This indicates to some extent that positive emotional state will enable individuals to obtain positive psychological capital. Based on the above research, this article proposes the following hypothesis:

**Hypothesis 4:** Psychological capital plays a mediating role in the relationship between family supportive supervisor behavior and employee creativity.

### The Mediating Role of Positive Emotions

Emotional spillover theory holds that emotions generated in a certain role may cause spillover effects, which can be divided into positive and negative aspects. Isen et al. (1987) and other scholars believe that positive emotions can effectively broaden their vision and thus have a clear awareness of the surrounding environment. Under the action of such broader awareness, employees can face various new ideas with an open mind and their creativity will be significantly improved. Kunze and Menges (2017) show that positive emotion can effectively improve employees’ work ability, hobbies, interests and struggle level. Yi et al. (2019) point out that positive emotions are connected with supportive behaviors of the outside world, which promote employees to have more beneficial behaviors. Secondly, positive emotions can improve an individual’s energy level and increase the likelihood of high involvement in another role. Gi et al. (2020) point out that high emotional state can activate recessive memory and inspire new thought.

Meanwhile, Fredrickson et al. (2003) proposes the expansion-construction theory of positive emotions. This theory believes that positive emotions can effectively expand the scope of individual immediate thinking-action, and provide long-term adaptive benefits for individual growth and development by constructing more durable personal resources such as intelligence, physiology, psychology, and society. Under the influence of positive emotion theory, Amabile and Pratt (2016) and other scholars conduct a series of studies on organizational support and employee creativity. These studies find that employees’ positive emotions are closely related to their subsequent creative thinking, that is, positive emotions have a positive effect on employees’ creativity. Madrid (2020) believe that individuals dominated by positive emotions can flexibly adjust their cognitive state and improve their thinking activity level of thinking. Conversely, individuals prefer conservative ideas. Gable and Dreisbach (2021) point out that people affected by positive emotions can remain rational in the face of risks and firmly believe that they can properly reach the target. Therefore, they will show excellent extended thinking in emergent problems. Park et al. (2021) points out that positive emotions caused by a certain role in an organization can promote employees’ investment in other roles and enhance their tolerance, thus improving performance or producing positive emotions. Based on the above research, this article proposes the following hypothesis:

**Hypothesis 5:** Positive emotions play a mediating role in the relationship between family supportive supervisor behavior and employee creativity.

Our model assumptions are presented in Table 1.

**TABLE 1:** Model assumptions.

Hypothesis	Research hypothesis
H1	Family supportive supervisor behavior positively affects employee creativity
H2	Family supportive supervisor behavior positively affects employees’ psychological capital
H3	Family supportive supervisor behavior employees’ positive emotions
H4	Psychological capital plays a mediating role in the relationship between family supportive supervisor behavior and employee creativity
H5	Positive emotions play a mediating role in the relationship between family supportive supervisor behavior and employee creativity
H6	Psychological capital positively affects positive emotions

**FIGURE 1:**
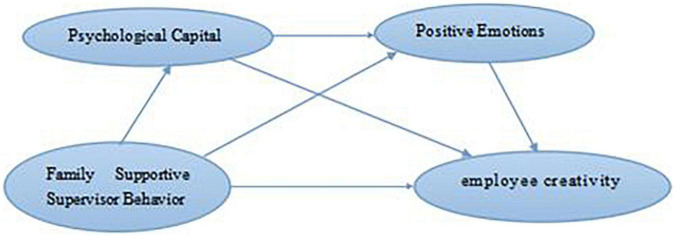
Research model.

Combined with research variables and research assumptions, the theoretical model of this study is specially constructed, as shown in Figure 1.

## Materials and Methods

### Participants and Procedure

This study focuses on the employees of Xinyu High Tech Industrial Park in Jiangxi Province with wide distribution of employees and obvious hierarchical differences. This study involves enterprises in regional pillar industries such as photovoltaic new energy, medicine and food. This study takes the industrial type and output value scale as the stratified sampling basis. Then this study carries on the stratified sampling to the existing 148 enterprises. In February 2021, after contacting the personnel department of the unit to be investigated in the industrial park, a questionnaire was issued to the employees to conduct research. After strict screening, 426 survey data were collected and 393 valid questionnaires were obtained, with an effective rate of 92.3%, excluding the samples with missed answers, incomplete answers and too little answer time.

The descriptive statistical results of the sample are as follows: 204 (51.91%) are men and 189 (48.09%) are women. In terms of educational background, 57 (14.5%) are senior high school and below, 108 (27.5%) are junior college, 185 (47.1%) are undergraduate, and 43 (10.9%) are graduate and above. In terms of age, 61 (15.5%) are 25 years old and below, 102 (26%) are 26–35 years old, 151 (38.4%) are 36–45 years old, 48 (12.2%) are 46–50 years old, and 31 (7.9%) are 51 years old and above. In terms of marital status, 96 (24.4%) were unmarried and 297 (75.6%) were married. In terms of the nature of units, 82 (20.9%) are state-owned enterprises, 76 (19.3%) are private enterprises, 30 (7.6%) are Sino foreign joint ventures, 21 (5.3%) are foreign-funded enterprises, 79 (30.1%) are government institutions and 105 (26.7%) are other units. In terms of length of service, 53 (13.5%) are within 1 year, 56 (14.2%) are 1–3 years, 77 (19.6%) are 4–7 years, 72 (18.3%) are 8–10 years, and 135 (34.4%) are more than 10 years.

### Measures

#### Family Supportive Supervisor Behavior

According to the scale developed by Hammer and other scholars, it is considered that FSSB is mainly reflected in employees’ perception of family supportive behavior expressed by leaders. The scale includes four items: “emotional support,” “instrumental support,” “role model behavior” and “creative work family management.”

##### Emotional Support

In the emotional support selection questionnaire, the items for “your supervisor is willing to listen to your problems between work and life,” “your supervisor takes time to understand your personal needs” and “your supervisor makes you feel very comfortable when talking about the conflict between your work and family.” Using Likert scale, options are “very disagree (= 1), disagree (= 2), neutral (= 3), agree (= 4), and very agree (= 5).”

##### Instrumental Support

In the instrumental support selection questionnaire, the items for “your supervisor and you have a very effective conversation on how to solve the conflict between work and family,” “if necessary, you can rely on your supervisor to help coordinate the conflict,” “when you have unexpected family needs, you can rely on your supervisor to ensure that your work responsibilities are handled” and “Your supervisor works efficiently with employees to creatively solve the conflict between work and family.” Using Likert scale, options as “very disagree (= 1), disagree (= 2), neutral (= 3), agree (= 4), and very agree (= 5).”

##### Role Model Behavior

In the role model behavior selection questionnaire, the items for “your supervisor is a good example of balancing work and family,” “your supervisor shows effective behavior on how to balance work and family” and “your supervisor shows how to win-win at work and family.” Using Likert scale, options are “very disagree (= 1), disagree (= 2), neutral (= 3), agree (= 4), and very agree (= 5).”

##### Creative Work Family Management

In the creative work family management selection questionnaire, the items for “your supervisor considers how to deal with work family conflict in order to benefit employees and the enterprise,” “your supervisor seeks suggestions on how to make it easier for employees to balance work and family needs,” “Your supervisor is very creative in reassigning work tasks and can help your department become a better work team” and “your supervisor can manage the whole department as a team to meet everyone’s needs.” Using Likert scale, options are “very disagree (= 1), disagree (= 2), neutral (= 3), agree (= 4), and very agree (= 5).”

#### Positive Emotions

In the positive emotion selection questionnaire, the items for “you rarely feel depressed and depressed,” “you rarely feel tired for no reason,” “you rarely find yourself irritable and unable to keep quiet,” “you rarely feel more irritable than usual,” “your mind is as clear as before,” “you are full of hope for the future,” “you feel it is not difficult to make decisions” “Things you used to do are still easy to do now,” “things you liked in the past are still like now,” “you think you are useful and needed,” “your mood is always high at work,” and “you are always full of vitality and energy at work” and other items. Using Likert scale, options are “very disagree (= 1), disagree (= 2), neutral (= 3), agree (= 4), and very agree (= 5).”

#### Psychological Capital

Psychological capital is divided into self-efficacy, hope, resilience and optimism. In the “self-efficacy” selection questionnaire, the items for “you believe you can analyze long-term problems and find creative solutions,” “when meeting with the management, you are confident in stating things within your scope of work” and “you believe you can creatively complete the goals you set.” In the “Hope” selection questionnaire, the items for “you can always think of many ways to get rid of the difficulties in your work,” “there are many solutions to any problem” and “you can think of many ways to achieve your current work goals.” In the “resilience” selection questionnaire, the items for “when you encounter setbacks at work, you can always recover from them and move on,” “at work, you will solve the problems you encounter anyway” and “you are usually calm about the pressure at work.” In the “optimistic” selection questionnaire, the items for “when you encounter neutral things at work, you usually expect the best results,” “you always see the bright side of things for your work” and “you are optimistic about what will happen in the future of your work.” Using Likert scale, options are “very disagree (= 1), disagree (= 2), neutral (= 3), agree (= 4), and very agree (= 5).”

#### Employee Creativity

In the employee creativity selection questionnaire, the items for “you will often adopt new methods to achieve work objectives,” “you will often put forward novel and feasible methods to improve work performance,” “you will often seek new technologies, processes, processes or ideas,” and “you will often put forward new suggestions to improve product quality or work quality.” Using Likert scale, options are “very disagree (= 1), disagree (= 2), neutral (= 3), agree (= 4), and very agree (= 5).”

### Reliability and Validity Test

Before the formal analysis of the sample data, the reliability and validity of the seven scales of emotional support, instrumental support, role model behavior, creative work and family management, positive emotion, psychological capital and employee creativity are tested. The Cronbach’s α coefficient of each variable is calculated by SPSS 23.0 software. The specific results are shown in Table 2 reliability analysis of variables.

**TABLE 2:** Reliability analysis of variables.

Variable	Cronbach’s α	Item
Emotional support	0.969	3
Instrumental support	0.952	4
Role model behavior	0.935	3
Creative work family management	0.945	4
Positive emotions	0.949	12
Psychological capital	0.937	12
Employee creativity	0.901	4
Population	0.913	42

It can be seen from the results in Table 2 that the Cronbach’s α coefficient values of all variables are between 0.801 and 0.969, exceeding 0.8, indicating that the sample data reliability of the questionnaire is good. From the overall Cronbach’s α coefficient value, it reached 0.913, far more than 0.8, indicating that the scale has a certain good reliability, which provides a basis for further research.

Secondly, the KMO value of each variable was calculated. The results are presented in Table 3 validity result analysis.

**TABLE 3:** Validity result analysis.

	KMO-value	Approximate chi-square	df	Significance
Emotional support	0.969	5436.096	91	0.000
Instrumental support	0.912	1253.091	31	0.000
Role model behavior	0.931	879.692	45	0.000
Creative work family management	0.839	957.971	56	0.000
Positive emotions	0.960	3445.991	66	0.000
Psychological capital	0.962	3605.293	66	0.000
Employee creativity	0.884	1104.991	10	0.000
Population	0.932	8234.720	1092	0.000

The data in Table 3 show that the corresponding probability *p*-value of the overall scale represented by each factor in the formal questionnaire is 0.000, which is less than the significance level a, and there is a significant difference. At the same time, the KMO values of all scales are higher than 0.8, and the validity of the questionnaire is good.

### Common Variance Analysis

Harman single factor method was used to test the common variance in the study, so that all measurement items were loaded on a common potential factor (Zhou and Long, 2004). The results showed that the model fitting was poor, CMIN/DF = 4.377, RMSEA = 0.093, CFI = 0.821, GFI = 0.539, AGFI = 0.491, NFI = 0.780, TLI = 0.812. The fitting index is much worse than the original model, so there is no serious common variance.

### Ethics Statement

This study was conducted in accordance with the Declaration of Helsinki, and the protocol was approved by the Ethics Committee (HREC) of the School of Economics and Management in East China Jiaotong University. The participants provided their written informed consent to participate in this study.

## Results

### Model Fit Degree Analysis

It can be seen from the model fitting index value in Table 4. The fitting results of the absolute fitting indexes of the structural equation model fitting degree can meet the evaluation standard, X^2^/DF, RMSEA fit ideal. From the relative fitting indexes, the results of NFI, RFI, IFI, TLI, and CFI fit well; NFI and RFI reached 0.9, and the model fitting effect is good. The simplified fitting indexes can meet the evaluation criteria, and the results of PNFI, PCFI, and PGFI fit well.

**TABLE 4:** Model fitting index values.

Statistical tests	Indicators	Evaluation criterion	Model results	Fitting
Absolute fitness index	CMIN/DF	<3.0	2.175	Ideal
	RMSEA	<0.08	0.055	Ideal
Value-added fitness index	NFI	>0.90	0.913	Ideal
	RFI	>0.90	0.935	Ideal
	IFI	>0.90	0.9.9	Ideal
	TLI	>0.90	0.935	Ideal
	CFI	>0.90	0.939	Ideal
Minimalist fitting index	PGFI	>0.50	0.731	Ideal
	PNFI	>0.50	0.835	Ideal
	PCFI	>0.50	0.878	Ideal

Overall, the structural equation model has good fitting.

### Testing of Hypotheses

Through the operation of structural equation model (SEM), the path coefficient level is analyzed to verify its effectiveness.

As shown by the model parameter estimation in Table 5, first, FSSB has a positive and significant impact on employee creativity (β = 1.031, *P* < 0.05), H1 is supported. Second, FSSB has a positive and significant impact on psychological capital (β = 0.454, *P* < 0.05), H2 is supported. Third, FSSB has a positive and significant impact on positive emotion (β = 0.457, *P* < 0.05), H3 is supported. Fourth, psychological capital has a positive and significant impact on employee creativity (β = 1.031, *P* < 0.05), H4 is supported. Fifth, positive emotion has a positive and significant impact on employee creativity (β = 0.454, *P* < 0.05), H5 is supported. Finally, psychological capital has a positive and significant impact on positive emotion (β = 0.521, *P* < 0.05), H6 is supported.

**TABLE 5:** Model parameter estimation.

Variable	Standardized path coefficients	S.E.	C.R.	*p*-value	Result
Psychological capital < ---family supported supervisor behavior	0.781	0.117	3.894	***	Supported
Positive emotion < ---family supportive supervisor behavior	0.682	0.235	2.137	0.033	Supported
Employee creativity < ---family supported supervisor behavior	0.830	0.198	2.165	0.024	Supported
Employee creativity < ---psychological capital	0.693	0.187	2.418	0.031	Supported
Employee creativity < ---positive emotions	0.721	0.197	2.314	0.021	Supported
Positive emotion < ---psychological capital	0.672	0.182	3.617	***	Supported

***p < 0.001.

**FIGURE 2:**
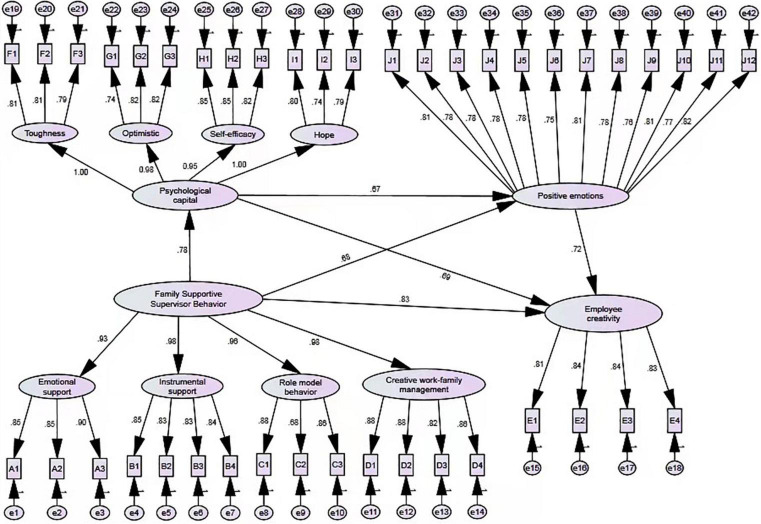
Statistical diagram of standardized path coefficient of structural equation model.

**FIGURE 3:**
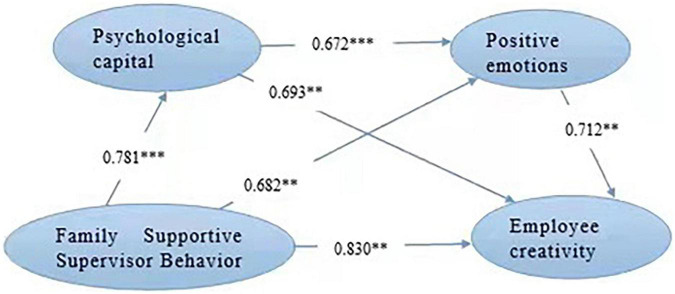
Structural equation modeling results. **p < 0.01, ***p < 0.001.

Figure 2 is the modified model diagram, and each path coefficient of the model is shown in the figure. The hypotheses were tested by using structural equation modeling (see Figure 3).

According to the revised model diagram, the following views are also confirmed:

First, FSSB has a direct positive impact on employee creativity. The path coefficient between the two is 0.83, indicating that for every unit of FSSB, employee creativity will increase by 0.83 units. The more significant the family supportive supervisor’s behavior, the higher the creativity of employees. According to the resource conservation theory, only by enriching employees’ internal resources can employees give better play to their creativity, and FSSB can effectively reduce the threat of resource loss faced by employees, enrich their internal resources, and have more resources to invest in innovation. Hypothesis 1 is tested.

Second, FSSB has a direct positive impact on psychological capital. The path correlation coefficient between the two is 0.78, indicating that for each unit of FSSB, psychological capital increases by 0.78 units. The more obvious the family supportive supervisor’s behavior is, the higher its psychological capital value is. From the perspective of resource conservation theory, psychological capital is a positive internal resource. FSSB can enable employees to enhance their sense of self-efficacy and enrich internal resources, so as to be more able to obtain resources, and psychological capital will be enhanced accordingly. Hypothesis 2 is tested.

Third, FSSB has a direct positive impact on positive emotion. The path coefficient between the two is 0.68, indicating that for each unit of FSSB, positive emotion increases by 0.68 units. The more obvious the family supportive supervisor’s behavior is, the higher its positive emotion is. According to the emotion spillover theory, the family support behavior of supervisors will have a positive spillover effect on employees, and then affect employees’ emotions, endogenous positive emotions. Hypothesis 3 is tested.

Fourth, psychological capital has a direct positive impact on employees’ creativity. The path coefficient between the two is 0.69, indicating that for every unit of employees’ psychological capital, employees’ creativity will increase by 0.69 units. The higher the value of employees’ psychological capital, the higher their creativity. From the perspective of social exchange theory, the family support behavior shown by leaders will breed employees’ willingness to repay the organization, produce a closer exchange relationship between employees and organizations, and then stimulate employees’ creativity. Hypothesis 4 is tested.

Fifth, positive emotions have a direct positive impact on employees’ creativity. The path coefficient between the two is 0.72, indicating that for every unit of positive emotion, employee creativity will increase by 0.72 units. The higher the positive mood of employees, the higher the burst of creativity. From the perspective of emotion spillover theory, the supportive behavior of leaders will have a certain positive spillover on employees, make employees have positive emotions, promote them to invest more in innovation, show expansive thinking and improve creativity. Hypothesis 5 is tested.

Sixth, psychological capital has a direct positive impact on positive emotions. The path coefficient between the two is 0.69, indicating that every unit of employees’ psychological capital increase, their positive emotions increase by 0.69 units. The richer the psychological capital of employees, the higher their positive emotions. Psychological capital has a direct positive impact on positive emotions. Hypothesis 6 is tested.

Seventh, psychological capital and positive emotion play a mediating role. In order to further test the mediating effect of psychological capital and positive emotion, this study uses Amos software and deviation corrected bootstrap confidence interval method, and the confidence level is set to 95%.

According to the bootstrapping mediation effects testing in Table 6, the following conclusions can be drawn: first, the bootstrap deviation correction confidence interval of the indirect effect of psychological capital on FSSB and employee creativity under 95% confidence is (0.086, 0.626), zero is not within this range, and the *p*-value is less than 0.05. It shows that psychological capital has a significant mediating effect between FSSB and employee creativity. At the same time, the bootstrap deviation correction confidence interval of the direct effect of psychological capital on FSSB and employee creativity is (0.508, 0.611), zero is not within this interval, and the *p*-value is less than 0.05, indicating that psychological capital plays a partial mediating effect between FSSB and employee creativity. Second, the bootstrap bias correction confidence interval of the indirect effect of positive emotion on FSSB and employee creativity under 95% confidence is (0.037, 0.232), zero is not within this interval, and the *p*-value is less than 0.05, indicating that the mediating effect of positive emotion between FSSB and employee creativity is significant. Moreover, the bootstrap deviation correction confidence interval of the direct effect of positive emotion on FSSB and employee creativity is (0.508, 0.611), zero is not within this interval, and the *p*-value is less than 0.05, indicating that psychological capital plays a partial mediating effect between FSSB and employee creativity. To sum up, according to the bootstrap test results, both Family supported supervisor behavior - > psychological capital - > employee creative behavior path and Family supported supervisor behavior - > positive emotion - > employee creative behavior path pass the significance test at the level of 0.01. It can be seen that the two intermediary paths are significant.

**TABLE 6:** Bootstrapping mediation effects testing.

Summary of the hypothesized path	Coefficient	Boot standard error	Bias-corrected 95% CI		Statistical significance
				
			LL	UL	
Gross effect Family supportive supervisor behavior- > employee creativity	0.535***	0.517	0.503	0.597	significant
Direct effect Family supportive supervisor behavior - > employee creativity	0.560***	0.524	0.508	0.611	significant
Indirect effect Family supportive supervisor behavior - > psychological capital - > employee creativity	0.303***	0.137	0.086	0.626	significant
Family supportive supervisor behavior - > positive emotion - > employee creativity	0.075***	0.068	0.037	0.232	significant

CI, confidence interval; LL, lower limit; UL, upper limit. ***p < 0.001.

### Comparison of Differences Between Multi-Group Models

The internal structures of all the groups were heterogeneous. Therefore, it was necessary to compare the differences between the groups of different ages and types of household registration to reveal the differences between different groups more intuitively and accurately. This study divided the sample into those 25 years old and below, 26–35 years old, 36–45 years old, 46–50 years old, and 51 years old and above as well as agricultural households, according to the age and the type of household registration.

Table 7 reflects the degree of fit between the different group models and the surveyed data. The empirical results show that the GFI indexes of the three models of those 25 years old and below, 36–45 years old and 46–50 years old meet the evaluation criteria, showing that the fitting indexes of the two models are ideal. However, in the two models for those 26–35 years old, and 51 years old and above, the AGFI value is slightly lower than the evaluation standard, showing that the fitting of the two models is good. The parameter values of the two models of agricultural household registration and non-agricultural household registration are within the reference range, showing that the two models fit perfectly. Therefore, the fitting levels models constructed in this study are acceptable.

**TABLE 7:** Comparison of parameters between different groups.

Variable	Different population	GFI	AGFI	RMSEA	TLI	Result
		>0.90	>0.90	<0.08	>0.90	
Different age groups	25 years old and below	0.921	0.926	0.020	0.932	Ideal
	26–35 years old	0.934	0.893	0.066	0.915	Preferable
	36–45 years old	0.922	0.917	0.073	0.910	Ideal
	46–50 years old	0.941	0.911	0.077	0.933	Ideal
	51 years old and above	0.925	0.897	0.053	0.927	Preferable
Type of account	Agricultural	0.927	0.916	0.063	0.923	Ideal
	Non-agricultural	0.945	0.923	0.071	0.939	Ideal

*“Ideal” means that the fitting index is within the reference value range; “good” means that the fitting index is not within the reference value range but is slightly lower or slightly higher.*

From the different age groups, we compared the effects and path coefficients between FSSB, employee creativity, psychological capital and positive emotions, as shown in Table 8. The coefficients in the Table 8 are standardized.

**TABLE 8:** Comparison of path standardization coefficient between different groups.

Path	Different age groups					Type of account	
	<25	26–35	36–45	46–50	>51	Agricultural	Non-agricultural
Psychological capital < –family supported supervisor behavior	0.51***	0.46***	0.25***	0.31***	0.17**	0.31***	0.56***
Positive emotion < —family supportive supervisor behavior	0.26***	0.25***	0.13*	0.27***	0.15*	0.18***	0.26***
Employee creativity < –family supported supervisor behavior	0.06	0.12	0.10	0.15	0.01	0.14***	0.08
Employee creativity < –psychological capital	0.16***	0.10*	0.09	0.13	0.12	0.11***	0.16***
Employee creativity < — positive emotions	0.01	0.13	0.08**	0.05	0.02	0.01	0.07***
Positive emotion < — psychological capital	0.08	0.08**	0.09***	0.07***	0.05	0.06***	0.09***

*p < 0.05, **p < 0.01, ***p < 0.001.

First, the influence coefficient of FSSB on Employee creativity in those 51 years old and above and 46–50 years old are greater than others. Those 25 years old and below’s FSSB has the smallest impact coefficient, which is only 0.17. The possible reason is that people aged 25 and younger are younger, mostly struggling and face fewer family problems, and are less sensitive to family support; while employees aged 46 and older need more family support to have the energy to provide more creativity.

Second, the influence of FSSB in those 25 years old and below and the 26–35 years old on Positive emotion and Psychological capital is greater than others. The possible reason is that young people are more likely to have emotional and psychological satisfaction with support, and they feel more positive emotions and psychological capital, which helps to increase their creativity.

Third, the FSSB of the non-agricultural household registration has a greater impact on Employee creativity and Positive emotion than the agricultural household registration. At the same time, the psychological capital and positive emotions of the non-agricultural household registration have a greater impact on Employee creativity than the agricultural household registration. The possible reason for this is that the urban population is more educated, more knowledge, and more concerned about support by the leadership. They are more competent for more complex and important jobs, and after achieving some work results, the overflow of psychological capital and positive emotions once again promotes them to exert more creativity.

## Discussion

This article analyses the descriptive statistical characteristics of the samples, establishes a structural equation model to explore the impact of family supported supervisor behavior and employee creativity, and uses the bootstrap method of deviation correction to test the mediating effect of psychological capital and positive emotion on family supported supervisor behavior and employee creativity. Through the research, the following basic conclusions are drawn: first, FSSB has an obvious positive impact on employees’ creativity. Based on modern social exchange theory and resource conservation theory, when supervisors show work family support and emotional identity to employees, the more individuals can feel the intangible support from the enterprise, and thus burst out creativity in order to achieve the enterprise goals. Second, family supportive supervisor behavior has a significant positive impact on psychological capital and positive emotion, and they xx play a mediating effect in the mechanism. Third, Employee creativity can be stimulated by promoting job engagement and mobility. Supervisor supportive behavior and job rotation can effectively improve employees’ ability to adapt to their careers, and help them accumulate relevant knowledge and technical ability in the field as well as the motivation of creative problem solving. Importantly, this approach can effectively improve employees’ creativity.

### Implication for Research and Practice

How to improve employees’ creativity is the focus of increasing attention from all walks of life. According to the conclusion of this article, the following suggestions are put forward:

First, supervisors provide incentives to encourage family support behaviors. Whether the supervisor shows the behavior of family support should be properly included in the assessment, and the guiding role of performance management should be given full play. Organizations should improve the enthusiasm of the supervisor to show the behavior from the aspect of the assessment system. At the same time, the organization needs to give proper tilt to the demonstrating and effective supervisors in personnel adjustments, salary increases and promotions, etc., in order to show how much the organization attaches to the effective implementation of this informal organization support behavior.

Second, the organization provides formal family support system, such as flexible working hours, paid leave, telecommuting, to meet staff work and family needs, supervisor as the agent of the organization, needs more flexible to meet the personalized needs of employees, provide emotional support, consider employee feelings, when employees need support, to communicate with them in a way to make employees feel comfortable.

Third, employees have different needs and perceptions of family support supervisor behavior. Supervisors should provide different family support supervisor behavior according to the personal characteristics of different employees. Supervisors can understand employees’ needs for family support from daily observation and communication. We can meet the psychological needs of the new generation of employees from the following points: expanding autonomy while enriching choice opportunities, giving positive feedback in time, paying attention to emotional support, creating a sense of belonging and superimposing psychological capital.

Fourth, managers should cultivate family supportive leadership and enhance the behavioral willingness to implement family support. This study shows that family supportive supervisor behavior has a significant positive effect on employee creativity, and organizational managers should focus on cultivating family supportive leaders. In the selection of leaders, we can choose leaders with family support tendency as much as possible. At the same time, special lectures were held for supervisors at all levels to make them aware of the importance of showing family supportive supervisor behavior to improve employees’ creativity and enhance their willingness to implement such behavior. In addition, Organizations should combine the corporate culture and rules and regulations to standardize leadership behavior and cultivate family support supervisors.

Fifth, we should pay attention to emotional support and take full advantage of psychological capital and positive emotion. Organization can build a harmonious organizational atmosphere, establish a high-level relationship between leaders and members, and create a cultural atmosphere of emotional support. Organizations can provide emotional support resources, strengthen humanistic care, give employees adequate psychological compensation, and promote positive spillover effects. The third is to provide instrumental support. Supervisors provide targeted support resources and services in daily management to help employees perform their work and family responsibilities. Fourth, attach great importance to example behavior, and show family support to employees in coordinating and balancing work and family relations.

### Limitations

First, the questionnaires of this study are completed by employees, which will cause certain common variance. In future, the leader employee paired survey should be adopted and supervisor evaluation should be combined with the employee self-evaluation.

Second, this article only considers the influence of the situational variable of supervisor support behavior, but does not consider the role of factors such as the personal characteristics, the personal characteristics and cultural differences. It can be included in the scope of investigation for in-depth analysis in future research.

Third, this article examines employees’ creativity from the individual level. However, there are many other influencing factors of employees’ creativity, including team level and organization level. In future, cross level analysis can be incorporated into the research of employees’ creativity influence mechanism.

## Data Availability Statement

The datasets presented in this article are not readily available because of privacy protection regarding the data of the participants. Requests to access the datasets should be directed to the corresponding author.

## Author Contributions

XZ and LJ designed the research and methodology, and put forward the policy recommendations. XZ provided guidance throughout the entire research process. HY and LJ compiled the literature. YW and LL revised and approved the manuscript. All authors contributed to the article and approved the submitted version.

## Conflict of Interest

The authors declare that the research was conducted in the absence of any commercial or financial relationships that could be construed as a potential conflict of interest.

## Publisher’s Note

All claims expressed in this article are solely those of the authors and do not necessarily represent those of their affiliated organizations, or those of the publisher, the editors and the reviewers. Any product that may be evaluated in this article, or claim that may be made by its manufacturer, is not guaranteed or endorsed by the publisher.
